# Neurological manifestations and etiological risk factors in patients hospitalized with COVID-19 in Turkey

**DOI:** 10.2478/abm-2022-0004

**Published:** 2022-02-28

**Authors:** Nuray Can Usta, Seyfi Kartal, Betul Onal Gunay, Cavit Boz

**Affiliations:** Department of Neurology, University of Health Science, Trabzon Kanuni Training and Research Hospital, Trabzon 61250, Turkey; Department of Anaesthesiology and Reanimation, University of Health Science, Trabzon Kanuni Training and Research Hospital, Trabzon 61250, Turkey; Department of Ophthalmology, University of Health Science, Trabzon Kanuni Training and Research Hospital, Trabzon 61250, Turkey; Department of Neurology, Karadeniz Technical University, School of Medicine, Trabzon, Turkey

**Keywords:** COVID-19, neurological manifestations, SARS-CoV-2, seizure, Turkey

## Abstract

**Background:**

Coronavirus disease 2019 (COVID-19) can affect the neurological as well as the respiratory system. Neurological manifestations may involve the central or peripheral nervous systems, or musculoskeletal system. Findings can range from mild presentations, such as headache and anosmia, to severe complications, such as stroke and seizure.

**Objectives:**

To evaluate the neurological findings and to determine etiological risk factors for mortality in patients hospitalized for COVID-19.

**Methods:**

Medical records of patients with COVID-19 who were hospitalized and sought neurological consultation between March 2020 and March 2021 at a reference pandemic hospital in Turkey were reviewed retrospectively in a cross-sectional study design.

**Result:**

We included data from 150 (94 male) patients. Their mean age ± standard deviation was 68.56 ± 16.02 (range 21–97) years. The patients were categorized into 2 groups according to any acute neurological event or progression of neurological disease. Ischemic cerebrovascular events, seizures, and encephalopathy were the most common acute neurological events, while deterioration in consciousness, epileptic seizures, and Parkinson disease were observed in those with progression of neurological disease. Abnormal neurological findings were found at a mean of 7.8 ± 9.7 days following COVID-19 diagnosis and 50 (a third of) patients died. A logistic regression model found that advanced age, increased Modified Charlson Comorbidity Index (MCCI) score, and prolonged duration of hospitalization were factors significantly associated with increased mortality; however, sex and day of abnormal neurological findings after COVID-19 diagnosis were not. Common conditions accompanying neurological events were hypertension, coronary artery disease–heart failure, and diabetes mellitus.

**Conclusion:**

COVID-19 may present with neurological symptoms in our Turkish patients and comorbidities are often present.

Coronavirus disease 2019 (COVID-19) is a multisystemic disease that can primarily affect the neurological system as well as the respiratory system [1, 2]. Severe acute respiratory syndrome coronavirus 2 (SARS-CoV-2) infection usually causes mild symptoms such as fever, cough, and weakness. Infection may also cause respiratory, renal, and heart failure resulting in mortality [2,3,4,5]. COVID-19 neurological manifestations may involve the central nervous system (CNS), peripheral nervous system (PNS), or musculoskeletal system [1, 6]. The neurological findings can vary from mild presentations, including headache and anosmia, to severe complications, including stroke and seizure [1, 6, 7]. One mechanism alone cannot explain such a wide range of neurological findings, suggesting that multiple mechanisms may be involved. Neurological findings are considered to appear due to neurotropism and the neuroinvasive capacity of SARS-CoV-2 [8, 9]. Support for the CNS involvement of the virus is the detection of the SARS-CoV-2 in cerebrospinal fluid [8]. The virus enters cells by binding to spike proteins on angiotensin-converting enzyme-2 (ACE-2) located on the capillary endothelium of the CNS, the lower and upper respiratory tract, and invades the neuronal tissues [10]. CNS involvement is either due to systemic vascular dissemination or local invasion from the cribriform plate on the ethmoid bone [11].

The incidence of autoimmune diseases can increase after COVID-19 [12, 13]. It may be that the virus triggers an autoimmune response through molecular mimicry, leading to the formation of autoantibodies and bystander activation [13, 14]. Some of the neurological complications may also be associated with the evolved immune response after COVID-19. A 36% incidence of neurological findings in patients with COVID-19 has been reported [6]. A prospective study in Turkey study found an incidence of 34.7% neurological findings in patients with COVID-19 patients [7]. Although symptoms such as fever, cough, and exhaustion due to COVID-19 infection were identified in the literature, data related to neurological findings are limited. The present study aimed to evaluate the neurological findings in patients hospitalized for COVID-19 and to determine possible etiological factors and risk factors for mortality.

## Methods

The medical records of patients who were hospitalized for COVID-19 and who sought neurological examination between March 2020 and March 2021 at our university hospital, which is the reference pandemic hospital in our region of Turkey for COVID-19 patients, were retrospectively reviewed. Approval of the Trabzon Kanuni Training and Research Hospital management and Local Ethics Committee for Non-Pharmaceutical Clinical Research (approval No. 2021/72, 2021 April 20) was obtained for the study, which was performed following the principles of the contemporary revision of the Declaration of Helsinki (64th WMA General Assembly 2013) and ICH Good Clinical Practice (GCP) Guidelines. Informed consent was specifically waived by the ethics committee because of the retrospective nature of the study. We used STROBE and RECORD statement guidelines for reporting the study [15, 16].

The patients had been diagnosed with COVID-19 by positive SARS-CoV-2 reverse-transcriptase polymerase chain reaction (RT-PCR) test result on a combined sample taken from throat and nasopharynx or negative SARS-CoV-2 RT-PCR (first, second, or third sample) along with characteristic findings on thorax computed tomography (CT) (characteristic lesions including diffuse ground-glass lesion bilaterally and/or consolidation zones). Exclusion criteria included the following: patients with suspected SARS-CoV-2 infection who were excluded from the diagnosis of COVID-19 by the results of the examination upon consultation request; patients who had no acute neurological event or no worsening of a current neurological condition; patients who were consulted in the emergency department without hospitalization; and patients who had undiagnosed pre-existing neurological disease before SARS-CoV-2 infection, and patients with incomplete data in their medical records.

Patients were then divided into 2 groups; patients with an acute neurological event (Group 1) and patients with progression of a current neurological condition (Group 2) depending on the neurological disease status.

Demographic data of patients, comorbidities, hospitalization duration in the ward and/or intensive care unit (ICU), discharge status (live–dead), and laboratory levels were recorded. The Modified Charlson Comorbidity Index (MCCI) was calculated.

### Statistical analysis

Statistical analysis was performed with IBM SPSS Statistics for Windows (version 22.0). Continuous variables are presented as mean ± standard deviation (SD), and categorical variables were presented as frequencies. Quantitative data distribution was determined with a Shapiro–Wilk test. A Student *t* test was used to compare 2 independent groups quantitatively. A χ^2^ test was used to compare qualitative data. Logistic regression analysis is used to determine the association of independent variables with one dichotomous dependent variable(mortality). *P* < 0.05 was considered significant.

## Results

**Figure 1:**
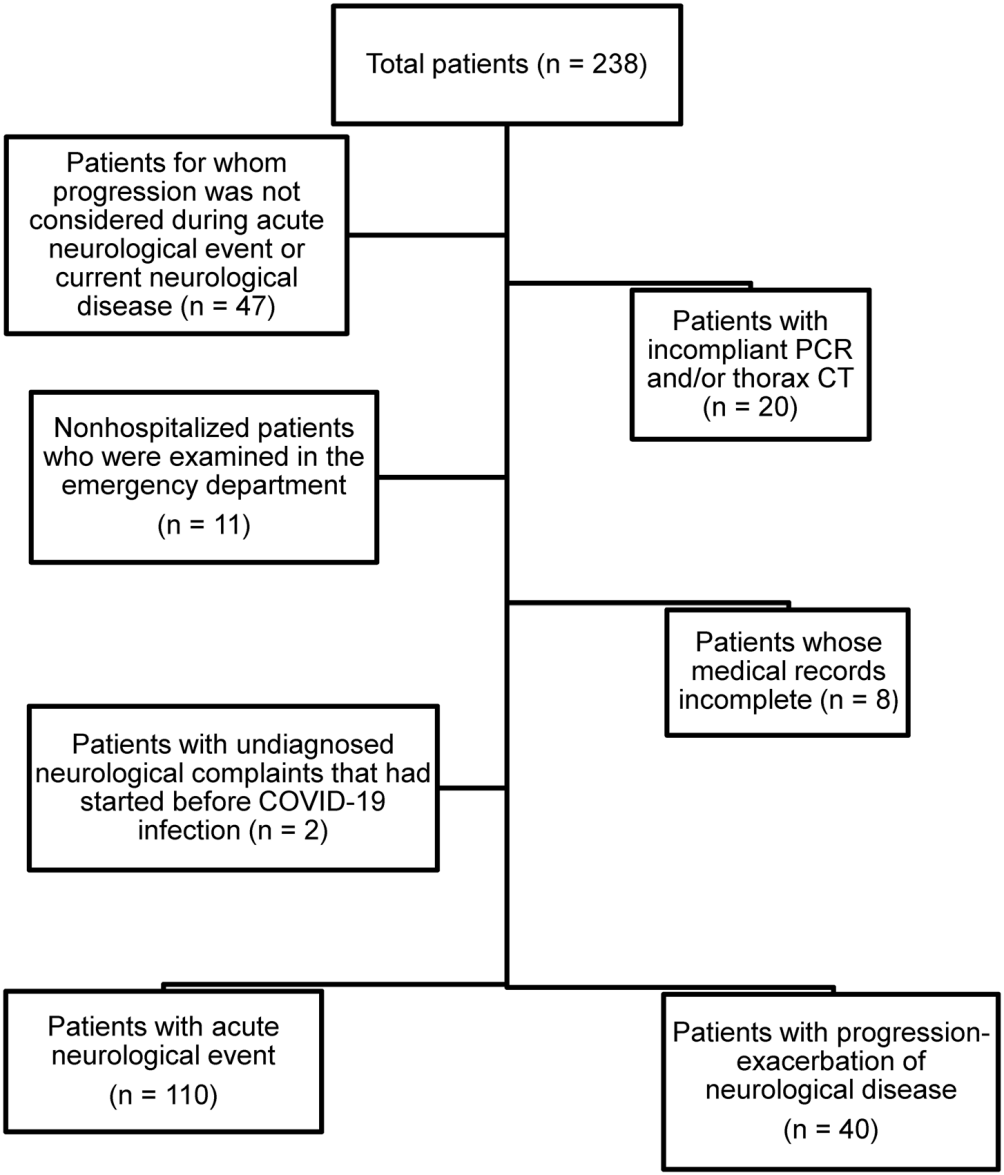
The number of patients excluded through exclusion criteria. COVID-19, coronavirus disease 2019; CT, computed tomography; RT-PCR, reverse-transcriptase polymerase chain reaction.

**Figure 2:**
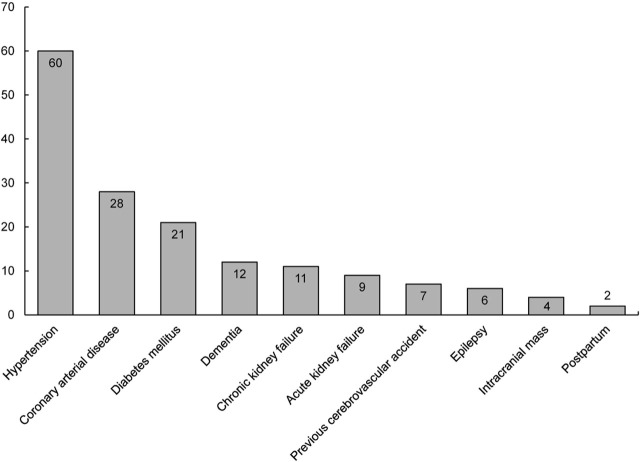
Frequency histogram indicating comorbidities in all patients studied. Coronary heart disease frequency includes heart failure.

Neurological consultation was requested for 238 of 4,295 patients who were hospitalized due to a diagnosis of COVID-19 between March 2020 and March 2021. After exclusions, we included data from 150 patients (94 male, 63%) in the present study (**Figure 1**). The mean age ± SD of the patients was 68.6 ± 16.0 years (range 21–97 years). The SARS-CoV-2 RT-PCR test result was positive in 140 (93%) patients and negative in 10 (7%) patients. Abnormal neurological findings were detected at a mean of 7.8 ± 9.7 days following RT-PCR or radiological test positivity. The average hospitalization duration of all patients was 15.2 ± 15.5 days. There were 110 patients in Group 1 and 40 patients in Group 2. There was no difference between these groups in terms of age, sex, or hospitalization in ICU (**Table 1**). The most frequent neurological events in Group 1 were ischemic cerebrovascular events (CVEs), seizure, and encephalopathy, while in Group 2, the most frequent neurological events were deterioration in consciousness, epileptic seizure, and Parkinson disease (**Tables 2 and 3**). While the mortality rate was 36.3% in Group 1, it was 25% in Group 2, but we found no significant difference overall (*P* = 0.19). Comorbid diseases are shown in **Figure 2**. Demographic and clinical data of the patients in Group 1 and Group 2 are summarized in **Tables 2 and 3**. There were 5 patients with undefined transient neurological findings such as visual hallucination (n = 2), anisocoria (n = 1), neuropathic pain (n = 1), and gait disturbance (n = 1).

**Table 1:** Demographic data of patients with an acute neurological event (Group 1) and patients with progression or exacerbation of a current neurological condition (Group 2)

	**Group 1 (n = 110)**	**Group 2 (n = 40)**	***P***
Age	67.7 ± 16.4	70.9 ± 15.0	0.28†
Female/male	46/64	10/30	0.06§
Service/ICU	57/53	21/19	0.94§

ICU, intensive care unit.

† Student *t* test.

§ χ^2^ test.

**Table 2:** Group 1, patients with an acute neurological event (n = 110)

**Event**	**Age (years)**	**Female/male**	**Dead/alive**	**COVID-19 disease severity (Mi/Mo/S)**	**MCCI**	**Day of neurological evaluation after COVID-19 diagnosis**
CVE ischemic	76 ± 9.9	12/23	17/18	15/0/20	5.5 ± 1.9	6.8 ± 8.3
Seizure	69.2 ± 12.7	8/9	9/8	8/0/9	4.9 ± 1.9	8 ± 9.3
Encephalopathy	74.6 ± 13.1	8/6	8/6	4/1/9	6.0 ± 2.9	6.5 ± 8.1
Dizziness	64 ± 20.3	4/8	2/10	9/1/2	3.3 ± 2.3	8.3 ± 8.8
Headache	51.2 ± 11.5	6/6	0/12	12/0/0	1.7 ± 1.3	3.8 ± 2.4
Myopathy/myositis	55.6 ± 28.5	1/3	2/2	0/1/3	2.8 ± 3.1	18 ± 16
SAH and CVE hematoma	60.6 ± 17.4	2/3	2/3	1/0/4	2 ± 2.8	6.7 ± 6.9
Agitation	53.5 ± 33.8	0/2	0/2	1/0/1	3 ± 4.2	37 ± 37
Facial paralysis	62 ± 1.4	1/1	0/2	1/1/0	3 ± 1.4	4 ± 4.2
Optic neuropathy	51.5 ± 23.3	1/1	0/2	1/0/1	3 ± 2.8	6.5 ± 2.5
Transient undefined	60.1 ± 18.1	3/2	0/5	3/1/1	4.8 ± 2.7	18 ± 21.1

Data are reported as mean ± standard deviation or frequency.

CVE, cerebrovascular event; COVID-19, Coronavirus disease 2019; F/M, female/male;Mi/Mo/S, mild/moderate/severe; MCCI, Modified Charlson Comorbidity Index; SAH, subarachnoid hemorrhage.

**Table 3:** Group 2, patients with progression in neurological disease (n = 40)

	**Age (years)**	**Female/male**	**Dead/alive**	**COVID-19 disease severity (Mi/Mo/S)**	**MCCI**	**Day of neurological evaluation after COVID-19 diagnosis**
Deterioration in consciousness	77.3 ± 9.7	5/19	8/17	12/2/10	6.1 ± 1.4	7.5 ± 7.2
Epileptic seizure	59.8 ± 19.3	0/5	1/4	3/0/2	4.6 ± 1.9	6 ± 3.53
Parkinson disease	77 ± 8.8	1/2	0/3	1/0/2	–	4.5 ± 2.12
Multiple sclerosis	53.3 ± 16.8	2/1	1/2	2/0/1	–	6.3 ± 4.9
Myasthenia gravis	59.3 ± 1.5	1/2	0/3	1/2/0	4 ± 1.4	6.5 ± 4.9
Motor neuron disease	76	0/1	0/1	0/0/1	6	7
Spastic tetraparesis	35	0/1	0/1	1/0/0	0	12

Data are reported as mean ± standard deviation or frequency.

COVID-19, coronavirus disease 2019; Mi/Mo/S, mild/moderate/severe; MCCI, Modified Charlson Comorbidity Index.

For patients in ICU (n = 72), the average duration of stay was 14.9 ± 15.6 days. The MCCI of all patients was 4.69 ± 2.27. Fifty (a third) of the 150 patients died. The logistic regression model for possible factors that may affect mortality found significance with advanced age (*P* = 0.005), increase in MCCI score (*P* < 0.001), and prolonged duration of hospitalization (*P* = 0.04); however, sex (*P* = 0.28) and time of abnormal neurological finding detection after COVID-19 diagnosis (*P* = 0.49) were not significantly related to mortality.

Comparison of laboratory test results between dead and alive patients found in deceased patients significantly higher levels of creatinine (1.8 ± 1.6 mg/dL versus 1.0 ± 1.3 mg/dL, *P* = 0.005), lactate dehydrogenase (LDH) (578.0 ± 457.6 IU/L versus 310.2 ± 125.9 IU/L, *P* = 0.001), ferritin (1,000.8 ± 771.3 μg/L versus 572.2 ± 551.9 μg/L, *P* = 0.003), procalcitonin (4.8 ± 13.4 versus 0.5 ± 2.5, *P* = 0.03), C-reactive protein (CRP) (153.1 ± 88.1 mg/L versus 82.9 ± 67.1 mg/L, *P* < 0.001), white blood cells (WBC) (12,456.2 ± 8,075.3 cells/μL versus 8,467.3 ± 4,228.5 cells/μL, *P* = 0.002), neutrophils (12.1 ± 10.4 cells/μL versus 6.7 ± 4.2 cells/μL, *P* = 0.001), International normalized ratio (INR) (1.19 ± 0.2 versus 1.09 ± 0.26, *P* = 0.04), and dimerized plasmin fragment D (D-dimer) (2,788.2 ± 4,749.1 μg/mL versus 1,212.8 ± 2,300.9 μg/mL, *P* = 0.03). Platelet cell count (205,750 ± 888,855 platelets/μL versus 255,187 ± 145,525 platelets/μL, *P* = 0.01) was significantly lower in deceased patients. We found no significant differences for other laboratory test results (**Table 4**).

**Table 4:** Laboratory test results for dead and alive patients

	**Dead patients (n = 50)**	**Alive patients (n = 100)**	***P* †**
Creatinine (mg/dL)	1.8 ± 1.6	1.0 ± 1.3	0.005
Sodium (mmol/L)	138.2 ± 6.6	136.6 ± 5.2	0.17
Potassium (mmol/L)	4.1 ± 0.7	4.2 ± 0.6	0.36
Aspartate aminotransferase (U/L)	81.0 ± 154.7	38.0 ± 39.2	0.06
Alanine aminotransferase (U/L)	48.4 ± 63.1	38.2 ± 38.1	0.31
LDH (IU/L)	578.0 ± 457.6	310.2 ± 125.9	0.001
CRP (mg/L)	153.1 ± 88.1	82.9 ± 67.1	<0.001
Procalcitonin	4.8 ± 13.4	0.5 ± 2.5	0.03
Ferritin (μg/L)	1,000.8 ± 771.3	572.2 ± 551.9	0.003
Creatine kinase (U/L)	500.8 ± 1,072.0	249.2 ± 616.9	0.24
White blood cell (cells/μL)	12,456.2 ± 8,075.3	8,467.3 ± 4,228.5	0.002
Neutrophils (cells/μL)	12.1 ± 10.4	6.7 ± 4.2	0.001
Lymphocytes (cells/μL)	0.9 ± 1.5	1.3 ± 1.42	0.73
Hemoglobin (g/dL)	11.7 ± 2.4	12.4 ± 2.1	0.08
Platelet cell count (cells/μL)	205,750 ± 888,855	255,187 ± 145,525	0.01
Prothrombin time (s)	14.2 ± 2.4	13.2 ± 2.9	0.052
Partial thromboplastin time (s)	28.2 ± 7.3	27.0 ± 8.5	0.41
INR	1.19 ± 0.2	1.09 ± 0.26	0.04
Fibrinogen (mg/dL)	530.5 ± 202.1	649.4 ± 773.1	0.34
D-dimer (μg/mL)	2,788.2 ± 4,749.1	1,212.8 ± 2,300.9	0.03

Data are reported as mean ± standard deviation or frequency.

CRP, C-reactive protein; D-dimer, dimerized plasmin fragment D; INR, International normalized ratio; LDH, lactate dehydrogenase.

† Student *t* test.

## Discussion

In the present study, we evaluated acute neurological complications and neurological disease progression in patients hospitalized with COVID-19. The 3 most common comorbid diseases accompanying neurological events were hypertension, coronary artery disease–heart failure, and diabetes mellitus; and several things could explain this association including increased prothrombotic risk, inflammation, or poor baseline cognition [5, 6]. Advanced age, increase in MCCI score, and prolonged duration of hospitalization were found to be associated with mortality.

We found an overall mortality rate of 33% for patients with COVID-19 who developed neurological complications. The mortality rates for Group 1 and Group 2 were 36.3% and 25%, respectively. In deceased patients, acute phase reactants (LDH, ferritin, procalcitonin, CRP, neutrophil), INR, and D-dimer were found to be significantly higher, and platelet cell count was found to be significantly lower, than in those who survived. Increased levels of acute-phase reactants, INR, D-dimer, and decreased platelet cell count may solely be due to COVID-19 infection, or the results of anticoagulant drug use, comorbid diseases, or secondary infections. Previous studies demonstrated that blood levels of D-dimer are higher in patients with neurological symptoms than patients without neurological symptoms [6, 17]. Platelet cell count and D-dimer levels were higher in patients with COVID-19 related pneumonia than in those with non-COVID-19 related pneumonia [18]. In accordance with the literature, D-dimer levels were also found higher in deceased patients, by contrast with the lower platelet cell count found in the present study.

Acute neurological findings in COVID-19 patients involve the CNS, PNS, and musculoskeletal system [6]. The most common neurological finding has been reported to be headache [17]. Although the incidence of headache varied between 11% and 14% in patients with COVID-19 [17], we found 8% of our patients had headache in the present study. This relatively lower incidence of headache in our study might be associated with the organization of medical treatment by the physician without consultation in hospitalized patients. We found that headache itself had no association with mortality.

Another important complication of CNS involvement is acute CVE (ischemic, hematoma, subarachnoid bleeding). In the present study, the incidence of an acute CVE in patients with COVID-19 was 23.3%. Incidence of CVE in COVID-19 patients has been reported as between 1% and 3% [6, 19, 20]. The increasing tendency toward coagulation downregulates natural anticoagulant mechanisms through inflammatory mediators; the reduction of the mortality rate by anticoagulant treatment supports the hypothesis of a deteriorated coagulation system [5, 21, 22].

Another complication detected in our study in CNS involvement was seizure. Generalized seizures have been reported in COVID-19 patients without any history of epilepsy [23]. The possible cause is triggering of neuronal hyperexcitability through receptor activation that depends upon the release of inflammatory cytokines, tumor necrosis factor α, and granulocyte colony-stimulating factor [23]. We detected a seizure rate of 16.6% in the present study. The incidence of seizures during COVID-19 has been reported as between 0.5% and 12.5% [24,25,26]. The higher rate of seizure in our present study might be related to the higher frequency of acute CVEs, as well as hypoxia and electrolyte imbalance. Detection of status epilepticus in 2 cases suggests that the virus may cause hyperexcitability during disease progress more than is currently considered. Because antiviral drugs such as lopinavir and ribavirin may trigger epilepsy, investigation of drugs used in case of epileptic seizure becomes important [27].

In the present study, the incidence of optic neuropathy among PNS complications was 1.3%. This incidence is consistent with reports of the incidence of visual loss as 1.4% [6]. Although many underlying causes may exist, inflammation and ischemia are considered possible causes for the development of optic neuropathy. Guillain Barré Syndrome (GBS) is also a PNS involvement together with optic neuropathy [28]. GBS was considered as the preliminary diagnosis in one patient; however, the diagnosis could not be confirmed as an electromyography (EMG) test could not be performed because of rapid deterioration of vital signs of the patient.

Mao et al. [6] identified skeletal muscle injury in the presence of myalgia and higher serum creatine kinase levels (>200 U/L) in musculoskeletal system injury during SARS-CoV-2 infection. Although the underlying mechanism of muscle injury was not clarified, increased proinflammatory cytokine-induced immune-mediated reaction was considered as the direct cause. These authors reported a rate of 19.3% for musculoskeletal system injury in patients with COVID-19. We observed a 2.6% rate of musculoskeletal system injury in the present study. The musculoskeletal system injury was found as a risk factor for mortality; however, the death of a 21-year-old postpartum patient without any recognized risk factor in the present study suggests that not all risk factors were revealed.

A major limitation of the current study is its retrospective design with a relatively low patient number. Common and mild symptoms during SARS-CoV-2 infection such as headache, dizziness, and loss of smell and taste could not be assessed. Furthermore, a neurological consultation may not have been requested because such symptoms may have been considered as a part of the natural progression of the disease. In the present study, the true incidence of symptoms and signs could not be revealed, because only patients hospitalized with COVID-19 and receiving treatment were evaluated. Neurological complications associated with COVID-19 such as transverse myelitis and sinus thrombosis were undetected in the present cross-sectional study, which might be a result of patients not being able to undergo further examinations because of their unstable condition.

## Conclusions

COVID-19 may present with a range of neurological symptoms. Neurological findings can either develop acutely or as a progression of neurological disease. An ischemic CVE was the most common acute neurological event, while deterioration in consciousness was observed in those with the progression of neurological disease. Although the overall mortality in our Turkish patients with the disease was low, the rate was higher in patients who develop neurological complications, and in patients with advanced age, high MCCI score, and prolonged hospitalization duration as consistent with other findings [29]. Differences in neurological complication rates compared with the literature may be associated with regional and genetic differences. Further multicentered prospective studies with longer monitoring periods are needed to determine the underlying pathogenesis of neurological disease in Turkish populations and the neurological findings that appear due to COVID-19.
